# Multiscale Ion-Electron Transport in 3D-Printed Hierarchically Porous Full Batteries

**DOI:** 10.3390/nano15211680

**Published:** 2025-11-05

**Authors:** Teng Wang, Lei Feng, Bohua Su, Xiaocong Tian, Yan Zhao

**Affiliations:** 1 School of Integrated Circuits, Wuhan University, Wuhan 430072, China; t.wang@whu.edu.cn; 2 School of Materials Science and Engineering, Wuhan University of Technology, Wuhan 430070, China; 359571@whut.edu.cn; 3 Faculty of Materials Science and Chemistry, China University of Geosciences, Wuhan 430074, China; 20151003636@cug.edu.cn; 4 College of Materials Science and Engineering, Sichuan University, Chengdu 610065, China

**Keywords:** 3D printing, full battery, multiscale transport, conductive network, hierarchical porosity

## Abstract

The rapid advancement of next-generation energy storage technologies demands advanced manufacturing strategies that offer structural precision, scalability, and compositional tunability. Three-dimensional (3D) printing has emerged as a transformative approach to constructing energy storage architectures. In this work, we report a 3D-printed LiCoO_2_//Li_4_Ti_5_O_12_ full battery featuring a hierarchically porous and conductive reduced graphene oxide-carbon nanotubes (rGO-CNTs) framework that enables desirable ion-electron transport. The resulting full cells exhibit a high capacity of 151.4 mAh g^−1^ at the rate of 0.1 C, superior rate performance, and outstanding cycling stability, maintaining 97.1% capacity after 3000 cycles. Furthermore, the fully printed cell successfully powers a digital stopwatch, demonstrating its practical applicability for devices. This study presents a structural and compositional study for constructing high-performance customizable 3D-printed batteries, advancing the digital manufacturing of next-generation energy systems.

## 1. Introduction

The rapid proliferation of portable electronics and electric vehicles has created an urgent demand for efficient and sustainable energy systems [1,2,3,4,5]. Traditional fossil fuels are gradually being replaced by renewable energy sources such as solar and wind; however, their intermittent nature necessitates reliable energy storage technologies. Among various energy storage devices, lithium-ion batteries (LIBs) have dominated the market owing to their high energy density, long cycling life, and stable operating voltage [6,7,8,9]. Nevertheless, with the growing pursuit of high power output, fast charging capability, and structural flexibility, conventional LIBs fabricated via slurry coating and lamination are approaching their performance limits [10,11,12,13,14].

Conventional electrode manufacturing methods often lead to dense structures with limited ion diffusion pathways. In particular, at a high mass loading level, the lack of interconnected macropores and multidimensional conduction networks results in severe polarization and poor rate capability under high current operation [15,16,17,18]. Three-dimensional (3D) printing, also known as additive manufacturing, has emerged as a transformative approach for constructing next-generation energy storage materials and devices [19,20,21,22,23]. By enabling programmable deposition of target materials, it allows the creation of hierarchically porous, mechanically robust, and spatially customizable electrode architectures with precisely controlled compositions and geometries [24,25]. These characteristics endow 3D-printed electrodes with high mass loading, efficient ion/electron pathways, and excellent design flexibility, making them promising candidates for integrated and high-performance energy storage systems [26,27,28,29,30].

Despite these advantages, achieving efficient ion-electron transport within thick or high-loading 3D-printed electrodes remains challenging [31,32]. When the electrode lacks hierarchical pore channels, ion transport through the tortuous network becomes sluggish, leading to incomplete utilization of active materials and limited rate capability. Constructing a multiscale porous framework that integrates macro-, meso-, and micropores may effectively overcome the transport limitations of dense electrodes [33,34,35,36]. In such a hierarchical architecture, macropores act as rapid ion highways, enabling efficient electrolyte penetration and minimizing diffusion resistance throughout the electrode thickness. Mesopores serve as intermediate channels that expand the electrode-electrolyte interfacial area, thereby facilitating charge storage and accelerating redox reactions [37,38,39,40]. Meanwhile, micropores provide abundant surface-active sites that enhance adsorption and contribute to pseudocapacitive behavior. Therefore, establishing an interconnected multiscale network that simultaneously facilitates ion diffusion and electron conduction is critical for enhancing both energy and power densities in 3D-printed lithium-ion batteries.

Herein, we present a 3D-printed LiCoO_2_//Li_4_Ti_5_O_12_ (LCO//LTO) full battery that integrates hierarchically porous architectures with a multiscale conductive framework composed of reduced graphene oxide (rGO) and carbon nanotubes (CNTs), achieving efficient ion-electron transport. Both the cathode and anode were fabricated via direct ink writing (DIW) using rheologically optimized inks. The hybrid rGO-CNTs framework forms a continuous conductive skeleton bridging LCO and LTO particles, while the 3D-printed lattice structure provides interconnected macropores for enhanced electrolyte infiltration and reduced diffusion tortuosity. The resulting full cell demonstrates a high specific capacity of 151.4 mAh g^−1^ at 0.1 C, excellent rate performance with only moderate capacity decay at 3 C, and remarkable cycling stability with 97.1% retention after 3000 cycles. Furthermore, the assembled printed full cell successfully powers a digital stopwatch, exhibiting immediate activation and stable operation over long durations, demonstrating the practical viability of the design for miniaturized power systems. These findings highlight a universal structural strategy for achieving high-performance, fully 3D-printed batteries that integrate tunable electrode architectures, robust conductive frameworks, and efficient multiscale transport pathways, paving the way for the development of customizable energy storage technologies.

## 2. Materials and Methods

### 2.1. Materials and Reagents

All the chemicals and materials were used without any further purification.

### 2.2. Fabrication of 3D-Printable Inks

The electrode inks were formulated using LiCoO_2_ (LCO, 99%, Alfa Aesar, Ward Hill, MA, USA) as the active material, rGO (Angxing Co., Ltd., Changzhou, China), CNTs (Xianfeng Nanomaterials Co., Ltd., Nanjing, China) and super P (Xianfeng Nanomaterials Co., Ltd., Nanjing, China) as the conductive additives, and polyvinylidene fluoride (Arkema, Paris, France) as the binder, with a mass ratio of 7:2:1. Within the conductive additives, the mass ratios of rGO to CNTs and rGO to Super P were both 1:1. Specifically, 500 mg of the mixed solid components were first dry-ground in an agate mortar for 10 min to ensure uniform mixing, followed by the gradual addition of 1.5 mL of N-methyl-2-pyrrolidone (NMP, 99%, Sigma-Aldrich, St. Louis, MO, USA) solvent. The resulting paste was further homogenized using a high-speed mixer (Hauschild SpeedMixer DAC 150.1, Hamm, Niederwinkling, Germany) at 2500 rpm for approximately 15 min to achieve a smooth and stable dispersion. The obtained slurry exhibited appropriate viscosity and thixotropy suitable for direct ink writing. Similarly, Li_4_Ti_5_O_12_ (LTO, Wanrun New Energy Technology Co., Ltd., Shiyan, China) anode inks were prepared following the same procedure to ensure consistent rheological behavior for both electrodes, enabling high-precision 3D printing of hierarchical lattice structures.

### 2.3. Electrode 3D Printing

First, the as-prepared LCO-G-C and LTO-G-C electrode inks were loaded into printing syringes equipped with stainless-steel nozzles of 250 μm inner diameter. The electrode geometries were digitally predesigned using parametric 3D modeling software and imported into the printer. A constant pneumatic pressure of 0.1–0.2 MPa was applied to extrude the ink smoothly onto clean quartz glass substrates at a controlled nozzle speed. By adjusting both printing pressure and speed, uniform grid-like lattices with various thicknesses were precisely fabricated.

### 2.4. 3D-Printed Battery Assembly

For half-cell measurements, CR2032 coin cells were assembled using the 3D-printed LCO-G-C electrodes as the cathode, lithium metal foil as the anode, a polypropylene separator, and an electrolyte consisting of 1 M LiPF_6_ dissolved in a 1:1 volume ratio mixture of ethylene carbonate and dimethyl carbonate with 5 vol% fluoroethylene carbonate (Duoduo Chemical Reagent Co., Ltd., Suzhou, China) additive. For full-cell configuration, identical CR2032 coin cells were fabricated by pairing the 3D-printed LCO-G-C cathode with a 3D-printed LTO-G-C anode of equal printed layers, separated by the same polypropylene membrane and infiltrated with the same electrolyte composition. For full-cell tests, the electrolyte volume was fixed at 100 μL per coin cell. All cells were assembled in an argon-filled glovebox with oxygen and moisture levels maintained below 0.1 ppm to prevent side reactions during cycling.

### 2.5. Structural Characterization and Electrochemical Measurements

The morphology and structural features of the printed electrodes were characterized using a JEOL-7100F field-emission scanning electron microscope (JEOL Ltd., Tokyo, Japan). The average filament width, layer spacing, and pore size distribution were determined by measuring multiple regions across the 3D-printed lattice to ensure structural uniformity. The crystalline structure of the obtained samples was analyzed via X-ray diffraction (Bruker D8 Advance, Bruker Corporation, Karlsruhe, Germany) in the 2θ range of 10–80° at a scan rate of 5° min^−1^, confirming phase purity and structural stability after printing. The specific surface area and pore-size distribution were obtained using the Brunauer–Emmett–Teller (ASAP 2460, Micromeritics Instrument Corp., Norcross, GA, USA) methods, respectively, to evaluate the mesoporous nature of the printed electrodes. Electrochemical measurements were carried out using a Neware CT-4008 battery test system (BTS 8.0, NEWARE Instrument Co., Ltd., Shenzhen, China). Galvanostatic charge–discharge (GCD) curves of LCO-based half and full cells were recorded at different current densities. Cyclic voltammetry (CV) was performed on a CHI 760E electrochemical workstation (Shanghai Chenhua Instrument Co., Ltd., Shanghai, China) at various scan rates to assess redox reversibility. Electrochemical impedance spectroscopy (EIS) was conducted over the frequency range of 10^5^–0.01 Hz with an amplitude of 5 mV to evaluate interfacial resistance and ion diffusion behavior. All electrode electrochemical measurements were performed in half-cell configurations. All measurements were performed at room temperature under ambient conditions.

## 3. Results and Discussion

**Figure 1:**
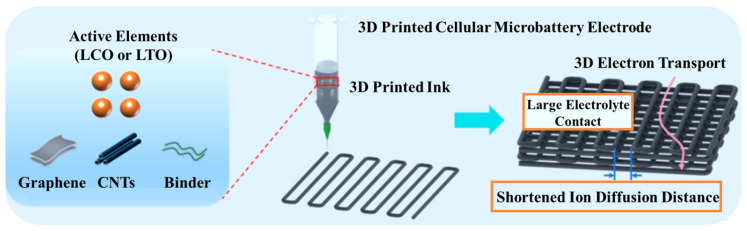
Schematic illustration of the 3D printing construction process of the electrode with a 3D network structure.

Figure 1 schematically depicts the design rationale for 3D-printed hierarchically porous electrodes used in this work. The fabrication workflow centers on three coupled stages: formulation of printable functional inks, precision DIW of periodic lattice architectures, and subsequent cell assembly. Such a workflow shifts the electrode design paradigm from passive, planar coatings toward actively engineered 3D geometries in which pore topology, strut spacing and layer stacking are digitally prescribed. This transition enables tunable electrolyte accessibility and controlled mass loading within a fixed footprint, prerequisites for translating intrinsic material capacity into practical areal energy density in compact devices. Beyond geometry, the electrode composition critically modulates charge-carrier transport. Here we designed composite inks that combine LCO active particles with an interconnected conductive scaffold built from rGO and CNTs, together with conventional binders. The hybrid rGO-CNTs framework serves two complementary roles: rGO provides broad-area, low-tortuosity planar sheets that promote lithium-ion accessibility and lateral electron delocalization, while CNTs form one-dimensional tubular bridges that reinforce pathways and mechanical integrity. When patterned into a periodic 3D lattice, these constituents generate a multiscale network that couples macroscale electrolyte channels with nanoscale electronic conduits. From the ion-transport perspective, the deliberate introduction of hierarchical porosity shortens effective diffusion distances and reduces tortuosity. The lattice provides large electrolyte-electrode interfacial area and rapid through-thickness pathways; concurrently, rGO sheets form accessible interstices at the particle scale that further facilitate Li^+^ ingress and homogenize local concentration gradients. Reduced tortuosity and increased electrolyte uptake are thus expected to suppress concentration polarization during high-rate operation and to narrow Warburg-type diffusion impedance observed in impedance spectroscopy.

Concerning electronic conduction, the hybrid rGO-CNTs network optimizes both long-range carrier mobility and interparticle contact resistance. The extended π-conjugation within rGO sheets facilitates in-plane electron transport across printed struts, while CNTs bridge inter-sheet gaps under mechanical deformation. As a result, printed electrodes exhibit lower charge-transfer resistance and improved rate capability compared with their particulate-only counterparts. The synergy between the two conductive motifs is particularly important for printed geometries where electronic pathways must traverse both printed filaments and interfilament voids.

**Figure 2:**
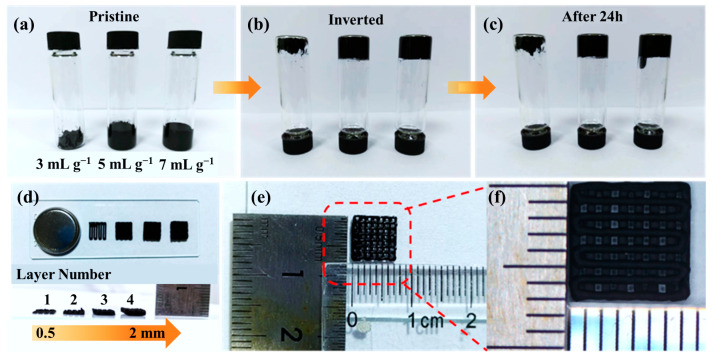
(**a**–**c**) Photographs of printable inks with different solvent contents after various inversion times; (**d**) optical images of 3D-printed battery electrodes with different printing layers; (**e**,**f**) dimensional view and magnified image of the printed electrodes.

Building on the design rationale in Figure 1, where digitally programmed lattices shorten ion paths and establish 3D electron percolation, the inks are verified reliable towards a stable written filament that is suitable for DIW. As shown in Figure 2a–c, inversion tests on inks with solvent-to-solid ratios of 3, 5, and 7 mL g^−1^ exhibited no phase separation at rest and no dripping upon inversion, indicating finite yield stress and rapid thixotropic recovery. Among these, the 5 mL g^−1^ formulation provided the best balance between extrudability and shape fidelity: 3 mL g^−1^ was overly viscous and interrupted flow, whereas 7 mL g^−1^ sagged and lost resolution. After 24 h the inks remained anti-sedimented, confirming storage stability and ensuring that the nozzle experiences the intended rheology. Consequently, the 5 mL g^−1^ ink was adopted for subsequent prints so that the programmed lattice could be rendered as a stable, hierarchically porous electrode. With rheology secured, we then assessed vertical build fidelity to guarantee that the targeted multiscale porosity is preserved during layer-by-layer deposition.

As displayed in Figure 2d, the printed filament thickness increases almost linearly from 0.5 mm to 2 mm as the number of printed layers increases, while the filament boundaries remain sharp with negligible lateral spreading. This vertical fidelity is critical for maintaining the designed pore-solid ratio, which governs both ionic diffusion and electronic transport. The preserved architecture ensures low tortuosity pathways for ion movement and uninterrupted 3D networks for electrons, fulfilling the dual-transport principle outlined earlier. To validate scalability, centimeter-scale lattices were further patterned as shown in Figure 2e,f. The deposited filaments exhibit highly uniform width and consistent spacing across the full print area, confirming high-resolution deposition and limited die-swell effects. This centimeter-level structural coherence coupled with sub-millimeter feature control establishes a robust framework for multiscale transport: large-area electrolyte contact promotes ionic accessibility, while the interconnected rGO-CNTs network supports rapid electron conductivity across micro- and macroscale domains. These geometric and structural foundations are essential for the subsequent evaluation of electrochemical responses in both half-cell and full-cell systems.

**Figure 3:**
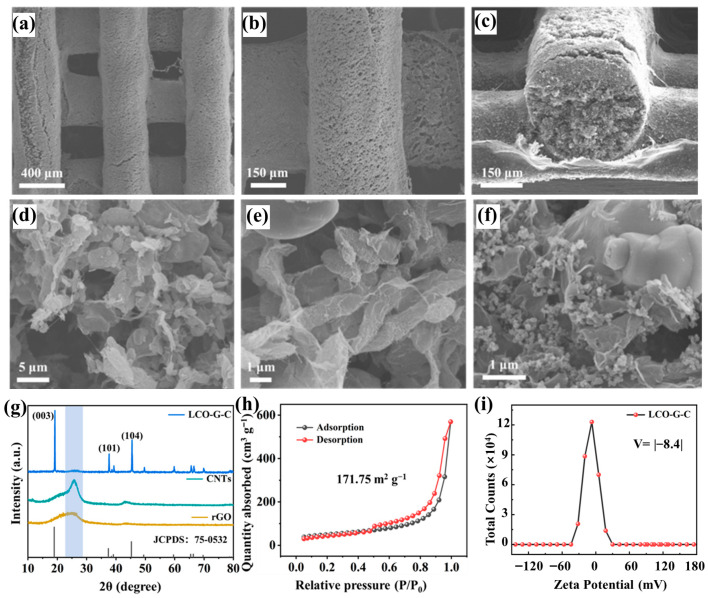
3D-printed electrode of (**a**,**b**) SEM images at different magnifications; (**c**) Cross-sectional image; (**d**,**e**) SEM images of the LCO-G-C electrode at different magnifications; (**f**) SEM image of the LCO-G-S electrode; (**g**) XRD pattern of the 3D-printed LCO-G-C electrode; (**h**) BET surface area plot; and (**i**) Zeta potential distribution of the 3D-printed LCO-G-C electrode.

Building upon the established 3D-printed lattice architecture, which ensures both geometric precision and microstructural continuity, further insights into the printed electrodes were gained through detailed morphological and compositional characterization. Low-magnification scanning electron microscope images reveal that the printed filaments form a highly ordered and well-registered lattice with uniform strand spacing (Figure 3a), and the smooth, ripple-free sidewalls (Figure 3b) indicate the rapid thixotropic recovery and high printing fidelity of the optimized 5 mL g^−1^ ink. The cross-sectional view (Figure 3c) shows each filament possessing a dense cylindrical core wrapped by a thin compact shell, while the inter-filament voids remain open. These interconnected channels penetrate vertically through the electrode, providing continuous diffusion pathways that effectively shorten ion transport distances and enhance electrolyte accessibility throughout the printed network. At higher magnification, the LCO-graphene-CNTs (LCO-G-C) electrode exhibits a hierarchically interwoven microstructure (Figure 3d,e). Wrinkled rGO sheets conformally coat LCO particles, forming lamellar conductive planes that promote in-plane electron transfer. Simultaneously, CNTs bridge adjacent grains and rGO domains, establishing long-range networks that maintain electrical continuity even under deformation. This multiscale conductive framework ensures rapid charge transport and uniform current distribution, mitigating polarization effects during cycling. In contrast, the LCO-graphene-Super-P (LCO-G-S) electrode displays evident carbon domain agglomeration and fewer conductive bridges (Figure 3f), resulting in discontinuous electron pathways and higher interfacial resistance. These structural distinctions demonstrate how the combination of rGO and CNTs yields a synergistic conductive network, integrating the planar conductivity of rGO with the one-dimensional percolation of CNTs to simultaneously facilitate ionic infiltration and electronic conduction. The resulting hierarchical porous architecture not only enhances the electrochemical kinetics but also underpins the superior transport behavior of the 3D-printed electrodes in subsequent performance evaluations.

X-ray diffraction (XRD) further validates that the 3D printing process maintains the intrinsic crystallinity of the active material. As shown in Figure 3g, the diffraction peaks of the printed LCO-G-C electrode correspond precisely to the layered LiCoO_2_ reference (JCPDS 75-0532), featuring distinct reflections at (003), (101), and (104) without the emergence of any secondary phases. This indicates that the DIW process followed by freeze-drying and curing does not induce lattice distortion or impurity formation, thereby preserving the cathode layered structure. A similar result is observed for the Super-P-based electrode (Figure S1), whose diffraction pattern aligns with the standard layered LiCoO_2_ structure, confirming that the integrity of the crystal framework is retained regardless of the type of carbon additive employed. To probe the pore characteristics crucial for mass transport, nitrogen adsorption–desorption measurements were conducted. The LCO-G-C electrode exhibits a type-IV isotherm with an H3-type hysteresis loop (Figure 3h), characteristic of mesoporous materials. The Brunauer–Emmett–Teller specific surface area reaches 171.75 m^2^ g^−1^, suggesting the presence of abundant mesopores originating from the rGO-CNTs conductive scaffold and the inter-filament voids of the 3D printed lattice. These mesopores not only provide additional electrolyte reservoirs but also serve as channels that homogenize ion flux and mitigate concentration gradients during high-rate cycling, collectively lowering the effective tortuosity of the electrode. In contrast, the LCO-G-S electrode (Figure S2) shows a similar type-IV isotherm but a markedly smaller BET surface area of approximately 57.25 m^2^ g^−1^ roughly one-third of that of LCO-G-C. This substantial difference underscores the superior structural accessibility and hierarchical porosity enabled by the rGO-CNTs hybrid framework, which enhances both electrolyte infiltration and ion diffusion kinetics. These findings reveal that the rationally designed multiscale porous network of the LCO-G-C electrode effectively bridges micro- and macro-scale transport, thereby laying a solid foundation for its enhanced electrochemical performance.

The reduced accessible surface area of the LCO-G-S electrode indicates a scarcity of mesoporous reservoirs and a correspondingly higher effective tortuosity for ion transport. This observation aligns well with the carbon agglomeration and discontinuous pore morphology revealed by SEM in Figure 3f. In contrast, the LCO-G-C electrode exhibits a more favorable colloidal behavior during ink preparation and extrusion, as evidenced by its zeta potential distribution centered at approximately −8.4 mV (Figure 3i). The moderately negative surface charge provides a balance between sufficient dispersibility and rapid gelation during the DIW process, ensuring structural uniformity upon deposition. This electrostatic condition, together with the steric hindrance imparted by the wrinkled rGO nanosheets, effectively prevents large-scale flocculation while enabling rapid recovery of a yield-bearing network after shear release. Such behavior guarantees consistent filament extrusion and smooth surface morphology, as previously observed in the SEM images. Conversely, the Super-P-based slurry displays a nearly neutral zeta potential (Figure S3), indicating weak electrostatic repulsion and a strong tendency toward particle aggregation during mixing and printing. This colloidal instability compromises the formation of a continuous conductive network and results in nonuniform pore structures after drying, thereby impeding both ionic and electronic transport. Collectively, these electrokinetic and morphological results emphasize that a well-balanced surface potential, aided by rGO-induced steric stabilization, is critical for constructing robust, homogeneous, and hierarchically conductive 3D-printed electrodes.

The supplementary characterization provides compelling evidence for why the rGO and CNTs hybrid network outperforms the super-P counterpart in the 3D-printed lattice electrodes. The LCO-G-C ink maintains a uniform dispersion and exhibits rapid recovery of a yield-bearing structure after shear release, which translates into a hierarchically porous, low-tortuosity framework once printed. This architecture verified by the high BET surface area and the interconnected pores visible in SEM and CT reconstructions facilitates fast electrolyte infiltration and establishes efficient, continuous pathways for both ion and electron transport. In comparison, the LCO-G-S electrode, though retaining an intact LiCoO_2_ crystal structure, presents a far smaller accessible surface area and less effective conductive percolation. The restricted porosity and discontinuous carbon domains result in higher charge-transfer resistance and hindered ionic diffusion, as corroborated by electrochemical impedance and rate-performance analyses. Collectively, these findings underscore that the multiscale interconnected carbon framework formed by rGO sheets serving as planar highways and CNTs bridging across neighboring grains within the DIW-printed lattice-is essential for achieving concurrent ion-electron transport. This synergistic configuration ensures rapid charge carrier mobility and structural robustness under high-rate cycling, thereby enabling superior stability and performance in 3D-printed full-cell systems.

**Figure 4:**
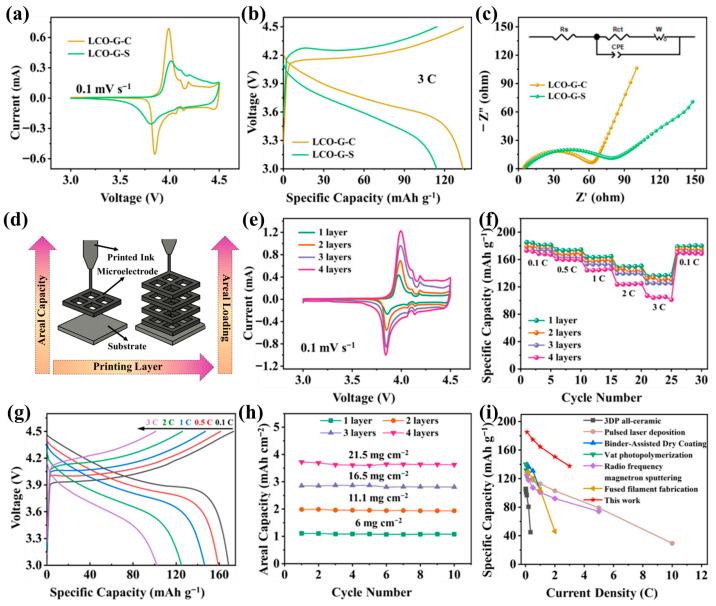
The electrochemical performances of the 3D-printed LCO-G-C and LCO-G-S electrodes. (**a**) Comparative CV curves; (**b**) GCD comparison; (**c**) EIS comparison; (**d**) Schematic illustration of the 3D printing process with increasing layer number; The 3D-printed LCO-G-C electrodes in half-cell with different printing layers of (**e**) CV curves; (**f**) Rate performance plots; (**g**) GCD curves of the four-layer LCO-G-C electrode; (**h**) Areal loading comparison; and (**i**) Ragone plot comparing 3D-printed LCO electrodes with other printing techniques.

As illustrated in Figure 4a, both LCO-G-C and LCO-G-S electrodes exhibit the characteristic Co^3+^/Co^4+^ redox pair near 3.9–4.2 V, indicating that the intrinsic electrochemical nature of LCO remains intact after DIW. Nevertheless, the LCO-G-C electrode demonstrates distinctly sharper and more symmetrical redox peaks with a reduced potential separation, signifying faster charge-transfer kinetics and lower polarization losses. This improvement arises from the interconnected rGO-CNTs framework, which enhances interparticle electrical continuity and accelerates Li^+^ intercalation/deintercalation dynamics. At a higher current density of 3 C (Figure 4b), the galvanostatic charge–discharge profiles further confirm these advantages. The LCO-G-C electrode maintains a stable and flat voltage plateau with minimal hysteresis, whereas LCO-G-S displays a pronounced IR drop and premature capacity decay, suggesting sluggish ion diffusion and poorer electrical contact. Electrochemical impedance spectroscopy (Figure 4c) quantitatively supports this observation: the Nyquist plots reveal a significantly smaller semicircle for LCO-G-C, indicating a lower charge-transfer resistance, and a steeper low-frequency slope, corresponding to a reduced Warburg diffusion impedance. These kinetic enhancements directly reflect the synergy between the hierarchically porous 3D lattice and the continuous rGO-CNTs conductive network, which together provide efficient and balanced transport channels for both ions and electrons. Figure S4 further highlights the rate capability differences between the two carbon architectures. When subjected to a stepwise current sequence from 0.1 C to 3 C and then returned to 0.1 C, the LCO-G-C electrode consistently delivers higher specific capacities with a notably smaller capacity decay compared to LCO-G-S. Upon reverting to 0.1 C, the LCO-G-C nearly recovers its initial capacity, indicating minimal kinetic hysteresis and excellent structural reversibility. This superior rate stability arises from the synergistic effects of the hierarchical porous lattice and the rGO-CNTs network, which provide efficient ion diffusion channels and uninterrupted electron transport pathways. Cycling tests (Figure S5) further confirm the structural and electrochemical robustness of 3D-printed LCO-G-C electrodes. At a constant current density, it maintains nearly 100% coulombic efficiency and retains 86.6% of its initial capacity after 5000 cycles, while the LCO-G-S electrode degrades significantly to 68.2%. After cycles, the 4-layer 3D-printed LCO-G-C electrode retains an intact macroscopic structure (Figure S6), and its hierarchical 3D porous framework together with the rGO-CNTs conductive network is preserved (Figure S7). This outstanding long-term cycling stability highlights the critical role of the multiscale conductive network, which simultaneously facilitates efficient ionic access and uninterrupted electron transport. The hierarchical 3D framework composed of interconnected rGO sheets and CNTs bridges, establishes a balanced dual-transport pathway that minimizes charge-transfer resistance and diffusion polarization. Such structural and electronic integrity enables 3D-printed electrodes to sustain high performance over extended operation, demonstrating their promise for durable and high-power energy storage systems.

The superior durability of the LCO-G-C electrode arises from two synergistic mechanisms. First, the 3D-printed lattice effectively alleviates concentration gradients and mechanical stress accumulation within thick electrodes, maintaining uniform ion flux and structural stability during extended cycling. Second, the rGO-CNTs conductive framework forms a robust, low-resistance interface that stabilizes the electrode-electrolyte boundary, suppressing parasitic side reactions and preventing repeated SEI reconstruction. The galvanostatic profiles after 5000 cycles at 3 C (Figure S8) vividly illustrate these effects. The LCO-G-C electrode retains a long and flat Co^3+^/Co^4+^ redox plateau with a narrow voltage gap, reflecting efficient charge-transfer kinetics and excellent capacity retention. In contrast, the LCO-G-S electrode exhibits an early voltage decline and a shortened plateau, indicative of higher polarization and structural degradation. The preserved plateau and reduced hysteresis of the LCO-G-C electrode are consistent with its lower charge-transfer resistance and weaker diffusion impedance, validating the multiscale transport design principle. Together, the hierarchical porous lattice and the interconnected carbon network enable simultaneous optimization of ionic diffusion and electronic conduction, thereby sustaining both power capability and cycling stability in 3D-printed full batteries.

Following the DIW-based structural blueprint (schematic in Figure 4d), the printing process was tuned to incrementally increase the number of layers while preserving the lattice geometry. This modular stacking strategy allowed a controlled enhancement of areal mass loading without compromising mechanical stability or diffusion efficiency. As shown in Figure 4e, cyclic voltammograms of the LCO-G-C electrodes with 1 to 4 layers retain distinct Co^3+^/Co^4+^ redox peaks, with only slight broadening as thickness increases. This behavior confirms that even at higher loadings, the 3D lattice efficiently facilitates ion transport and mitigates polarization. The corresponding rate performance (Figure 4f) further validates the structural integrity of the printed electrodes. Across current densities from 0.1 C to 3 C, all samples deliver stable capacities with minimal degradation, and upon returning to 0.1 C, the specific capacities nearly recover to their initial values-demonstrating outstanding reversibility and diffusion uniformity. Representative GCD profiles of the 4-layer electrode (Figure 4g) exhibit long, flat voltage plateaus, revealing excellent electrochemical kinetics and fast Li^+^ intercalation even under high loading conditions. Quantitatively, the areal loading can be tuned from 6 to 21.5 mg cm^−2^ (Figure 4h), accompanied by nearly linear gains in areal capacity, highlighting effective utilization of the additional active mass and the high structural fidelity of the printed framework. More importantly, the coexistence of macropores between filaments and the embedded mesoporous rGO-CNTs network ensures low tortuosity and continuous ion/electron pathways, thus preventing the severe rate decay commonly observed in thick LCO electrodes. When benchmarked against other fabrication techniques (Figure 4i), including all-ceramic 3D printing [41], Pulsed Laser Deposition [42], Binder-Assisted Dry Coating [43], photopolymerization [44], sputtering [45], and Fused Filament Fabrication [46], the 3D-printed electrodes occupy a superior region of the Ragone-type plot. They simultaneously achieve high gravimetric energy and power densities, underscoring the advantage of the multiscale transport mechanism enabled by the interconnected rGO-CNTs network and the digitally engineered porous architecture.

**Figure 5:**
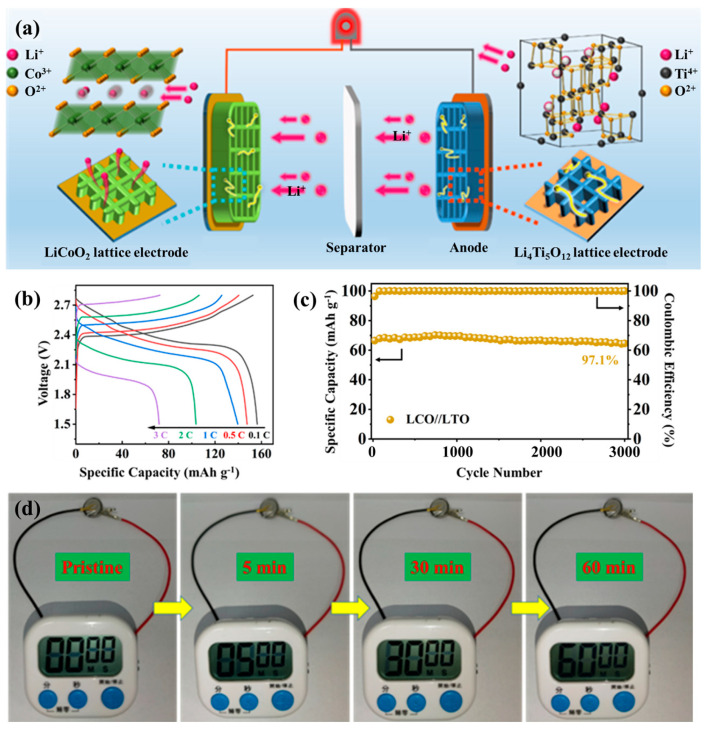
The 3D printed LCO//LTO full cell of (**a**) Schematic illustration of the working principle; (**b**) GCD curves at different current rates; (**c**) Cycling performance over 3000 cycles; and (**d**) Demonstration of the stopwatch powered by the 3D-printed full battery.

Building upon the optimized ink formulation and hierarchical microstructure engineering discussed above, the practical applicability of this 3D-printed architecture was evaluated through the assembly of a fully printed LCO//LTO full cell. Commercial Li_4_Ti_5_O_12_ (LTO) powder was incorporated with rGO and CNTs to develop a percolated conductive framework, forming a printable LTO-G-C ink via direct ink writing. When paired with the LCO-G-C cathode, the resulting LCO//LTO configuration offers well-matched electrochemical characteristics, making it an ideal platform to evaluate ion-electron transport within hierarchically porous electrodes. The full battery assembly is schematically illustrated in Figure S9. In this process, both the LCO- and LTO-based inks were directly printed into ordered 3D structures, followed by the placement of a separator between the two electrodes. The electrolyte was then introduced. The stacked assembly was encapsulated using compatible shells to complete the integrated full cell. This 3D configuration significantly enhances the electrode-electrolyte contact area, shortens Li^+^ transport pathways, and ensures continuous 3D electronic connectivity through the rGO-CNTs conductive framework. Such a design successfully translates the multiscale ion-electron transport strategy from the material to the device level. As depicted in Figure 5a, the working principle of the 3D-printed LCO//LTO cell relies on the synergy between structural hierarchy and compositional design. The LCO cathode lattice provides abundant electrolyte-accessible surfaces and ultrashort Li^+^ diffusion distances, while the integrated rGO-CNTs framework ensures rapid and uniform electron conduction across the printed filaments. On the anode side, the printed LTO lattice enables highly reversible Li^+^ insertion and extraction, maintaining structural stability throughout cycling. Coupled via a separator and fully infiltrated by electrolyte, the two electrodes establish a continuous multiscale transport network, where ions migrate efficiently through open macropores, and electrons percolate seamlessly through the conductive carbon skeleton.

A two-layer 3D-printed LCO-G-C cathode was paired with a two-layer LTO-G-C anode to construct a full cell operating within 1.5–2.8 V. The cyclic voltammetry curve at 0.1 mV s^−1^ (Figure S10) shows distinct lithiation and delithiation peaks with minimal potential separation, signifying highly reversible redox behavior and stable interfacial kinetics. The galvanostatic charge–discharge profiles (Figure 5b) further demonstrate the kinetic superiority of the hierarchical 3D architecture. The cell exhibits flat and symmetric voltage plateaus across 0.1–3 C, while the voltage hysteresis grows only slightly with increasing current density, confirming reduced tortuosity and low interfacial impedance in the printed lattice network. As illustrated in the rate performance tests (Figure S11), the full cell delivers specific capacities of 151.4, 148.9, 139.6, 105.2, and 72.9 mAh g^−1^ at 0.1, 0.5, 1, 2, and 3 C, respectively, and the capacity nearly fully recovers when the rate returns to 0.1 C, reflecting the mechanical robustness and structural integrity of the printed lattice. Electrochemical impedance spectroscopy (Figure S12) reveals a low charge-transfer resistance of 14.68 Ω and a steep low-frequency tail, which correspond to fast Li^+^ diffusion through the interconnected porous channels and continuous electron conduction along the rGO-CNTs framework. Collectively, these results confirm that the fully 3D-printed system achieves efficient ion-electron transport and excellent high-rate performance. Long-term cycling tests (Figure 5c) further demonstrate outstanding durability. After 3000 cycles, the LCO//LTO full cell retains 97.1% of its initial capacity with a nearly constant Coulombic efficiency, underscoring the mechanical resilience of the 3D lattice and the zero-strain nature of the LTO anode. The lattice-based structure prevents interfacial delamination and electrolyte depletion, ensuring continuous ionic and electronic pathways throughout extended cycling. This stability highlights the capability of the 3D-printed multiscale framework to sustain high-rate operation and long-term reliability in practical energy-storage devices.

To demonstrate device-level applicability, the assembled 3D-printed LCO//LTO full cell was utilized to power a digital stopwatch, as shown in Figure 5d. The stopwatch showed instantaneous activation and consistent operation for extended durations, captured at 5, 30, and 60 min. This demonstrates the strong power output and stable voltage delivery of the 3D-printed full cell. This successful demonstration bridges the gap between material-level optimization and practical device functionality, illustrating how the multiscale ion-electron transport network effectively translates into sustained, real-world performance. In conjunction with the preceding rate and cycling analyses, this result confirms that the designed hierarchically porous and electronically continuous structure not only supports efficient charge transport under laboratory conditions but also maintains reliability during continuous operation. The seamless synergy between the 3D-printed lattice architecture and the rGO-CNTs interconnected framework validates the proposed strategy as a universal and scalable pathway for the on-demand fabrication of all-3D-printed high-performance full batteries.

**Figure 6:**
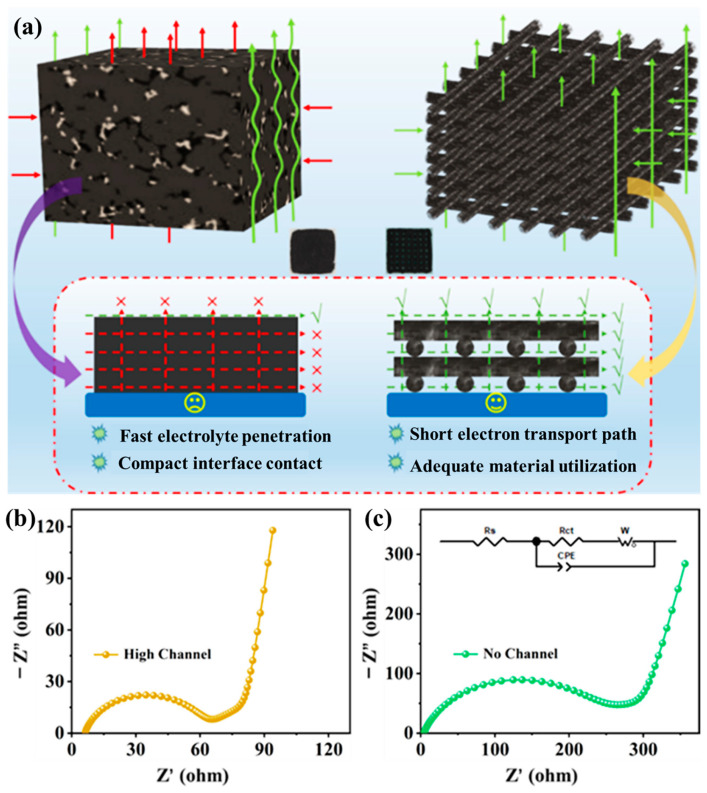
(**a**) Schematic illustration comparing charge-carrier transport in a conventional slurry-cast thick electrode and a 3D-printed high-loading electrode; EIS fitting of (**b**) the 3D-printed high-loading electrode; and (**c**) the conventional slurry-cast high-loading electrode.

Figure 6a provides a direct comparison between charge carrier transport in a conventional slurry-cast thick electrode and that in the 3D-printed high-loading LCO lattice. In the printed configuration, rGO sheets intertwined with CNTs form a hierarchically porous, percolated framework. Micron-scale surface pores on each filament accelerate electrolyte infiltration, while the interconnected through-plane lattice channels reduce Li^+^ diffusion distances and expose abundant active sites. Simultaneously, the carbon network ensures continuous 3D electron pathways, promoting compact electrode-electrolyte contact and high active-material utilization. In contrast, the conventional slurry-cast thick electrode lacks ordered channels, resulting in hindered electrolyte penetration and increased tortuosity, which lead to sluggish ion transport and uneven reaction fronts.

These distinct structural features are reflected in the electrochemical impedance spectra. As shown in Figure 6b, the Nyquist plot of the 3D-printed four-layer electrode exhibits a significantly lower charge-transfer resistance (Rct ≈ 55.35 Ω) and a reduced low-frequency Warburg slope, indicating enhanced Li^+^ diffusion and faster interfacial kinetics within the lattice framework. Conversely, the slurry-cast thick electrode (Figure 6c) presents a much higher Rct (237.9 Ω) and a pronounced diffusion tail, consistent with longer ion diffusion paths and limited electrolyte access. These findings confirm that the hierarchically porous, electronically interconnected structure effectively mitigates polarization by coupling rapid ionic infiltration with short and continuous electronic pathways. Consequently, the high-loading 3D-printed LCO electrodes maintain efficient charge transport even at increased thickness, establishing the foundation for high-areal-capacity and durable full-cell systems.

## 4. Conclusions

In conclusion, this work demonstrates a comprehensive multiscale ion-electron transport study in 3D-printed hierarchically porous full batteries. By developing optimized rGO/CNTs-based inks, we successfully constructed stable, shape-preserving lattices that translate nanoscale electronic percolation into macroscale electrode architectures. The printed LCO-G-C electrodes exhibit uniform pore distribution, high specific surface area, and low tortuosity, leading to superior electrochemical performance compared with conventional slurry-cast and super-P-based counterparts. Systematic characterization confirms the preservation of crystal integrity, enhanced ion diffusion, and continuous electron pathways across the hierarchically porous structure. At the device level, pairing the 3D-printed LCO//LTO full cell results in high reversible capacity, excellent rate performance, and 97.1% capacity retention over 3000 cycles. The device also demonstrated practical stability by reliably powering a digital stopwatch for extended durations, highlighting its real-world applicability. Overall, this research establishes a universal design framework that links ink rheology, multiscale architecture, and transport optimization. The proposed strategy offers a versatile route for scalable fabrication of high-loading, structurally integrated 3D-printed batteries and provides a solid foundation for advancing next-generation energy storage devices toward intelligent, on-demand, and high-performance applications.

## Data Availability

Dataset available on request from the authors.

## Supplementary Materials

The following supporting information can be downloaded at: https://www.mdpi.com/article/10.3390/nano15211680/s1, Figure S1: XRD pattern of the 3D-printed LCO-G-S electrode; Figure S2: BET surface area plot of the 3D-printed LCO-G-S electrode; Figure S3: Zeta potential distribution of the 3D-printed LCO-G-S electrode; Figure S4: Comparative rate performance of 3D-printed LCO-G-C and LCO-G-S electrodes at various rates; Figure S5: Comparative long cycles of 3D-printed LCO-G-C and LCO-G-S electrodes; Figure S6: Photographs of the 3D-printed LCO-G-C electrode in half-cell before and after charge-discharge cycles; Figure S7: SEM image of 3D-printed LCO-G-C electrode after cycles; Figure S8: Comparison of GCD performance of 3D printed LCO-G-C and LCO-G-S electrodes; Figure S9: Schematic diagram of the assembly of a 3D-printed full battery; Figure S10: CV plot of a 3D-printed LCO/LTO full cell; Figure S11: Rate performance plot of a 3D-printed LCO/LTO full cell; Figure S12: EIS fitting plot of a 3D-printed LCO/LTO full cell.
