# Supplementary material for: Multiscale Ion-Electron Transport in 3D-Printed Hierarchically Porous Full Batteries
Source: Nanomaterials (Basel). 2025 Nov 5;15(21):1680. doi: 10.3390/nano15211680 (PMC12608586; doi:10.3390/nano15211680)
Supplement: Supplementary file 1 [file nanomaterials-15-01680-s001.zip › nanomaterials-3960312-supplementary.pdf]

nanomaterials

MDPI

Article

Multiscale Ion-Electron Transport in 3D-Printed Hierarchically Porous Full Batteries

Teng Wang [1], Lei Feng [2], Bohua Su [3], Xiaocong Tian [2,3,*] and Yan Zhao[1,4,*]

[1] School of Integrated Circuits, Wuhan University, Wuhan, 430072, P. R. China
[2] School of Materials Science and Engineering, Wuhan University of Technology, Wuhan, 430070, P. R. China; xctian@whut.edu.cn
[3] Faculty of Materials Science and Chemistry, China University of Geosciences, 430074 Wuhan, P. R. China.
[4] College of Materials Science and Engineering, Sichuan University, Chengdu, 610065, P. R. China; yan2000@whu.edu.cn
* Correspondence: yan2000@whu.edu.cn, xctian@whut.edu.cn

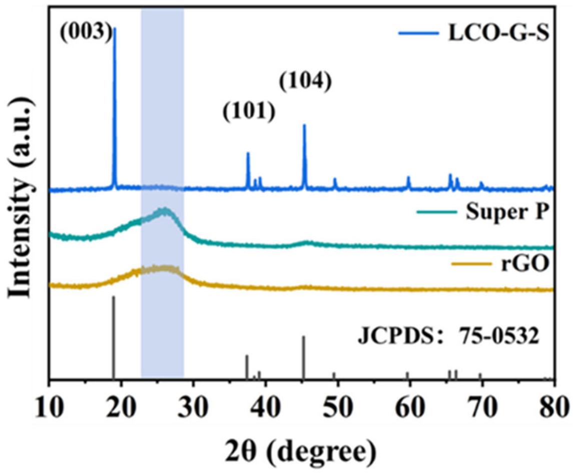

Figure S1. XRD pattern of the 3D-printed LCO-G-S electrode.

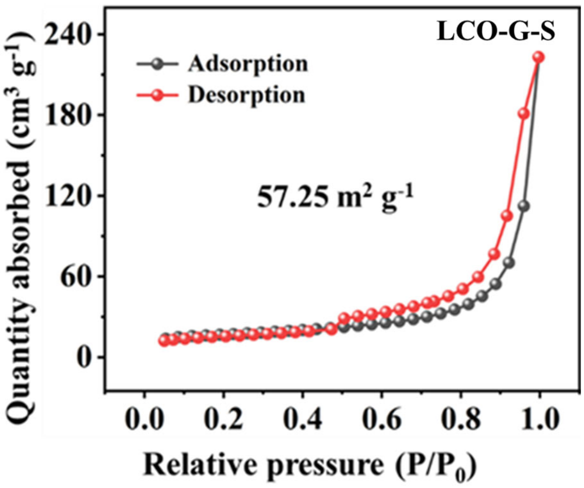

Figure S2. BET surface area plot of the 3D-printed LCO-G-S electrode.

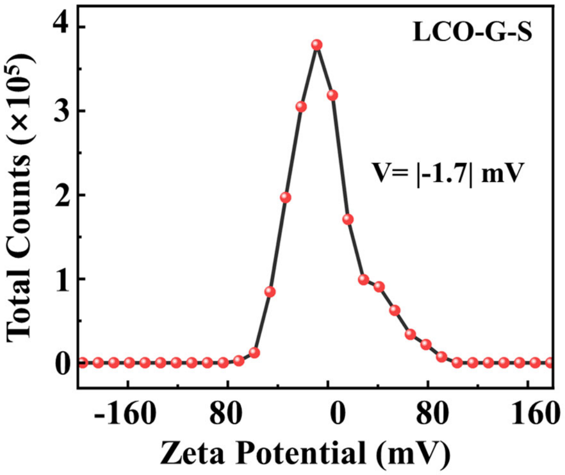

Figure S3. Zeta potential distribution of the 3D-printed LCO-G-S electrode.

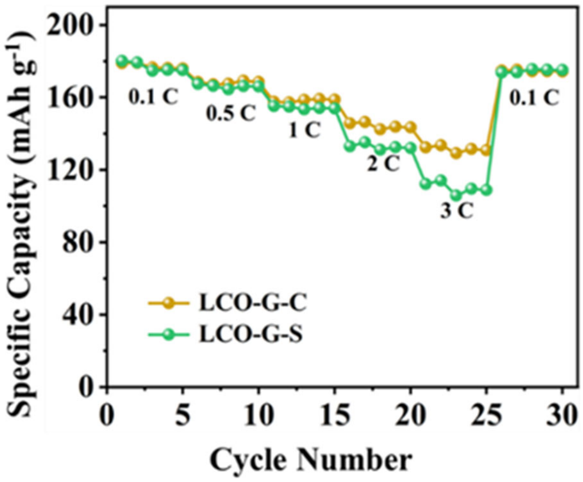

Figure S4. Comparative rate performance of 3D-printed LCO-G-C and LCO-G-S electrodes at various rates.

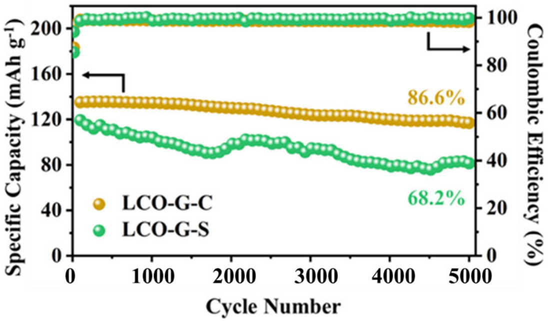

Figure S5. Comparative long cycles of 3D-printed LCO-G-C and LCO-G-S electrodes.

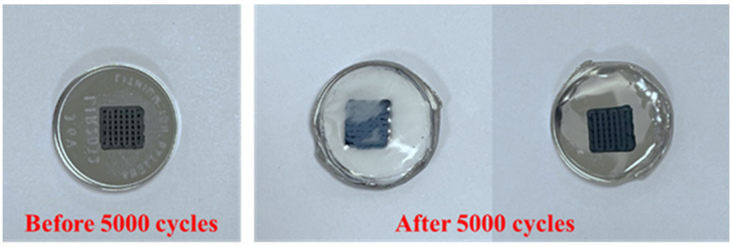

Figure S6. Photographs of the 3D-printed LCO-G-C electrode in half-cell before and after charge-discharge cycles.

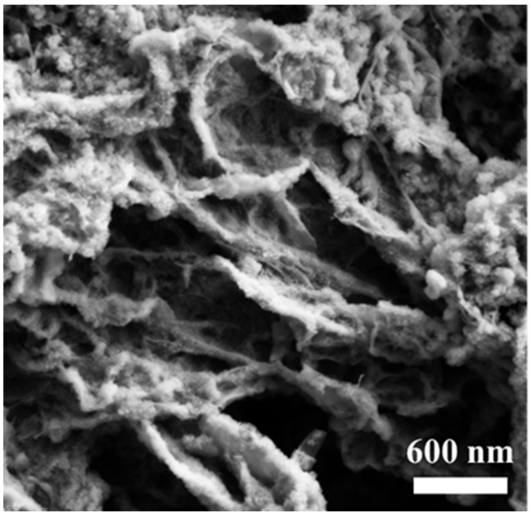

Figure S7. SEM image of 3D-printed LCO-G-C electrode after cycles.

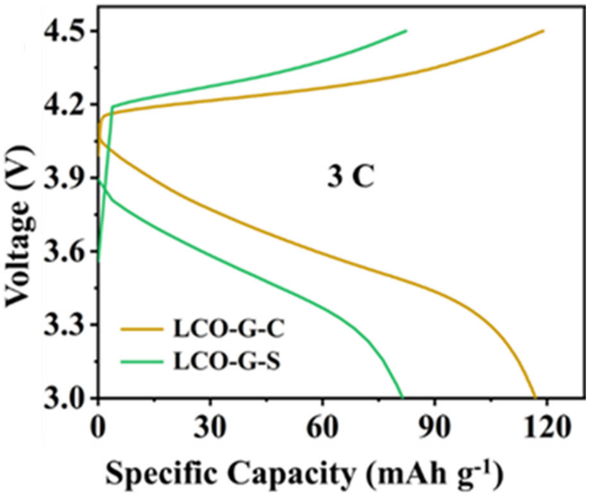

Figure S8. Comparison of GCD performance of 3D printed LCO-G-C and LCO-G-S electrodes.

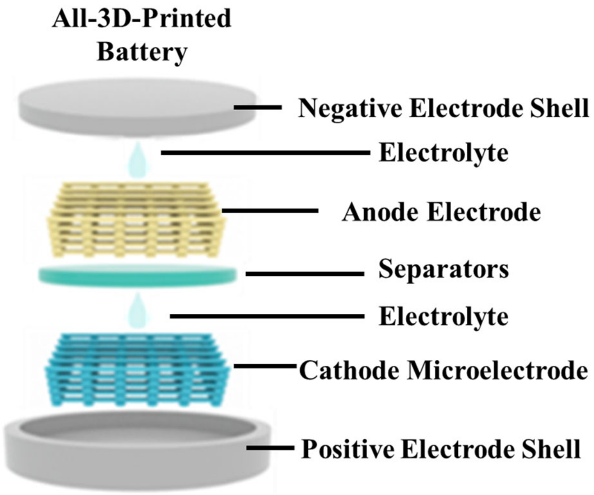

Figure S9. Schematic diagram of the assembly of a 3D-printed full battery.

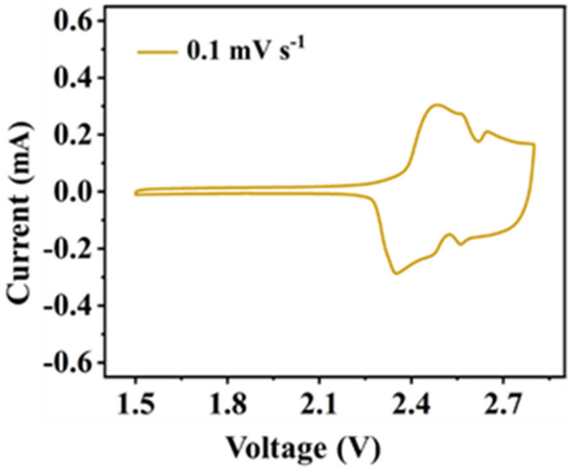

Figure S10. CV plot of a 3D-printed LCO/LTO full cell.

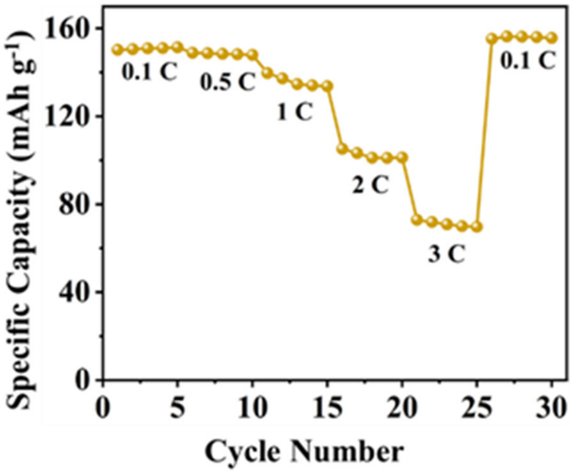

Figure S11. Rate performance plot of a 3D-printed LCO/LTO full cell.

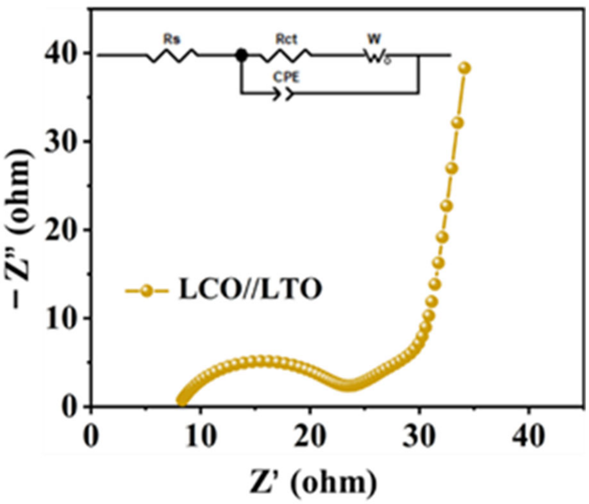

Figure S12. EIS fitting plot of a 3D-printed LCO/LTO full cell.
